# Ketogenic diet administration to mice after a high-fat-diet regimen promotes weight loss, glycemic normalization and induces adaptations of ketogenic pathways in liver and kidney

**DOI:** 10.1016/j.molmet.2022.101578

**Published:** 2022-08-20

**Authors:** Souad Nasser, Thomas Solé, Nathalie Vega, Thierry Thomas, Aneta Balcerczyk, Maura Strigini, Luciano Pirola

**Affiliations:** 1 South Lyon Medical Faculty, INSERM Unit 1060, Lyon 1 University, Pierre Benite, France; 2 INSERM, U1059 SAINBIOSE, UdL/UJM Saint-Etienne, University Hospital CHU, Saint-Etienne, France; 3 Department of Molecular Biophysics, University of Lodz, Faculty of Biology and Environmental Protection, Lodz, Poland

**Keywords:** Ketogenesis, Ketogenic diet, β-hydroxybutyrate, Histone PTMs, HMGCS2, ANOVA, analysis of variance, BHB, β-hydroxybutyrate, CD, chow diet, DEXA, Dual Energy X-Ray absorptiometry, HDAC, histone deacetylases, HFD, high fat diet, HMGCS2, Hydroxymethylglutaryl-CoA synthase 2, KD, ketogenic diet, PTM, post-translational modification

## Abstract

**Objective:**

The ketogenic diet (KD), characterized by very limited dietary carbohydrate intake and used as nutritional treatment for GLUT1-deficiency syndromes and pharmacologically refractory epilepsy, may promote weight loss and improve metabolic fitness, potentially alleviating the symptoms of osteoarthritis. Here, we have studied the effects of administration of a ketogenic diet in mice previously rendered obese by feeding a high fat diet (HFD) and submitted to surgical destabilization of the medial meniscus to mimic osteoarthritis.

**Methods:**

6-weeks old mice were fed an HFD for 10 weeks and then switched to a chow diet (CD), KD or maintained on a HFD for 8 weeks. Glycemia, β-hydroxybutyrate (BHB), body weight and fat mass were compared among groups. In liver and kidney, protein expression and histone post-translational modifications were assessed by Western blot, and gene expression by quantitative Real-Time PCR.

**Results:**

After a 10 weeks HDF feeding, administration for 8 weeks of a KD or CD induced a comparable weight loss and decrease in fat mass, with better glycemic normalization in the KD group. Histone β-hydroxybutyrylation, but not histone acetylation, was increased in the liver and kidney of mice fed the KD and the rate-limiting ketogenic enzyme HMGCS2 was upregulated – at the gene and protein level – in liver and, to an even greater extent, in kidney. KD-induced HMGCS2 overexpression may be dependent on FGF21, whose gene expression was increased by KD in liver.

**Conclusions:**

Over a period of 8 weeks, KD is more effective than a chow diet to induce metabolic normalization. Besides acting as a fuel molecule, BHB may exert its metabolic effects through modulation of the epigenome - via histone β-hydroxybutyrylation - and extensive transcriptional modulation in liver and kidney.

## Highlights

• In mice fed a high fat diet, the dietary switch to a ketogenic diet causes weight loss and loss of fat mass.
• Glycemic normalization is superior than observed in mice fed a chow diet.
• Ketogenic diet induces mild ketosis, and β-hydroxybutyrylation on histone H3 lysines.
• Upregulation of rate limiting ketogenic protein HMGCS2 is observed in kidney.
• Ketogenic diet may be a transitory nutritional intervention to favor weight loss.

## 1 Introduction

Ketogenic diets, consisting of the almost complete elimination of dietary carbohydrates, induce the body to rely on ketone bodies as primary energy source [1]. Ketone bodies, of which the quantitatively major species is β-hydroxybutyrate (BHB), are mainly produced hepatically from fatty acids precursors, and represent the major energetic source for the brain [2], heart, kidney [3] and other organs under conditions of carbohydrate deprivation.

In addition to its role of energetic substrate, BHB acts as an anti-inflammatory molecule [4], via inhibition of the of the NLRP3 inflammasome [5], and as a regulator of the epigenetic transcriptional state of chromatin by acting as a modulator of histone deacetylases (HDACs) [6,7]. BHB is also the substrate moiety for histone β-hydroxybutyrylation, a novel protein post-translational modification (PTM) - taking place on histone and non-histone proteins - associated to gene expression [8,9]. The participation of BHB in the regulation of gene transcription and inflammation, together with its capability to prevent senescence of vascular cells [10] and enhance their proliferative potential [11] may contribute to its positive effects on metabolism.

Somehow paradoxically, feeding on an energy-dense ketogenic diet is akin to starving or caloric restriction, two other nutritional states relying on the mobilization of stored fat depots once carbohydrate availability becomes scarce: in both cases, BHB will serve as a fat-derived fuel molecule substitute for glucose [12].

In specific circumstances, the administration of a ketogenic diet has been proven to be of clinical benefit, such as in pharmacologically intractable epilepsy [13] or in GLUT1 deficiency syndromes [14]. The feasibility of feeding on a ketogenic diet, associated with the metabolic similarities that the ketogenic diet shares with starving or caloric restriction, promoted the hypothesis that the uptake of a ketogenic diet may be beneficial and contribute to improved metabolic health and ageing. In mice, long term administration of a ketogenic diet enhanced longevity and “health span” [15]. From the clinical standpoint, the effect of ketogenic diet is complex and sometime opposite effects are observed. On the one hand multiple studies show that KD is clinically useful to alleviate epileptogenic seizures in pharmacologically refractory epileptic patients [13,16] and is recommended in mood disorders treatment [17], on the other hand it was found that a KD aggravates neurodegeneration in mice with induced mitochondrial DNA toxicity in the forebrain [18]. Also, while the KD ameliorates the clinical state of malignancies of the central nervous system in rodent models and humans [[19], [20], [21]], a firm proof from a randomized clinical trial is still missing.

In particular, a KD may have therapeutic value in situations where metabolic disturbance is associated with increased risk and severity of a given pathology, as in the highly prevalent case of obesity-compounded osteoarthritis [22]. Within this context, we investigated in mice the metabolic and epigenetic effects of a KD administered to osteoarthritic animals rendered obese by previous administration of a high-fat-diet. We report here that a KD induces: (i) weight loss, (ii) a better glycemic normalization than a chow diet, (iii) histone β-hydroxybutyrylation, (iv) remodeling of the expression of ketogenic and ketolytic genes/proteins both in the liver and kidney and (v) strong upregulation of the rate-limiting ketogenic enzyme HMGCS2 in the kidney, suggesting that the kidney may be a ketogenic organ.

## 2 Materials & methods

### 2.1 Animal protocols

Thirty 5-weeks old healthy C57Bl6/J male mice fed on chow diet were obtained from Charles River Laboratories (Saint Germain Nuelles, France). Animal experimental procedures were conducted in accordance with institutional guidelines for the care of laboratory animals and accepted by the French Ministry of research under the study protocol #12127-2017110911058255 v2.

After 1-week acclimatization, 6-weeks old mice were submitted to a high fat diet regimen (D12492, SSNIFF Spezialdiäten, Soest, Germany) for 10 weeks. After the 10 weeks HFD regimen, all animals were submitted to a surgical destabilization of the medial meniscus (DMM) to induce osteoarthritis. After DMM, mice were randomized into three groups by a blinded observer, receiving (i) a continued high fat diet (HFD group, empty circles, n = 10), (ii) a chow diet (CD group, SAFE A03 diet, U8200G10R, Augy, France; gray circles, n = 10) or (iii) a ketogenic diet (KD group, EF R/M with 80% fat, SSNIFF Spezialdiäten, Soest, Germany; black circles, n = 10) for 8 weeks. Macronutrients composition of the commercial diets is reported in Supplementary Table 1. All nutritional regimens were given *ad libitum*.

**Figure 1:**
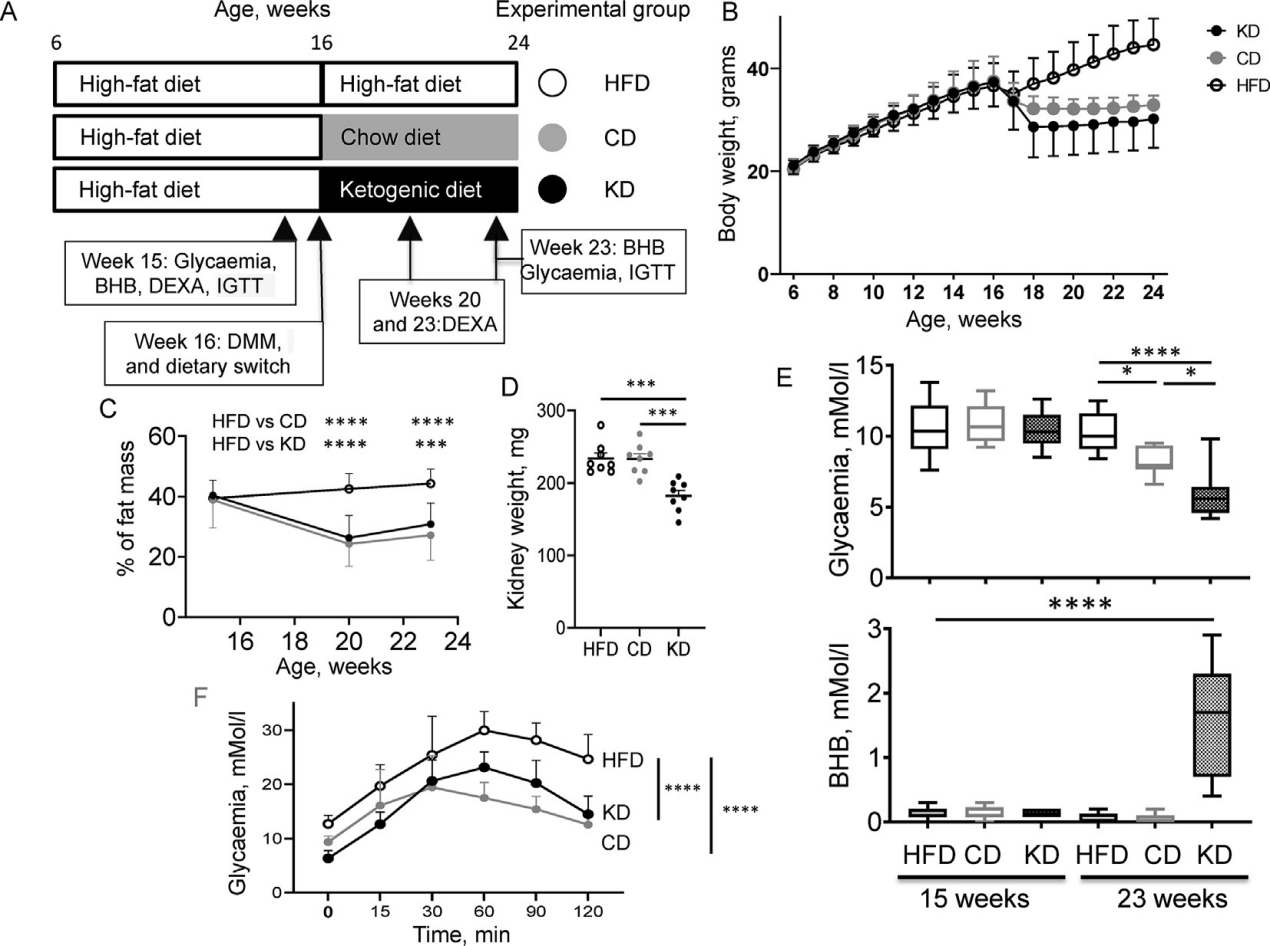
Experimental protocol diagram and metabolic parameters. (A) Schematic representation of the study protocol. Groups are defined by empty circles (HFD throughout the study), grey circles (CD, switch to a chow diet) and black circles (KD, switch to a ketogenic diet). (B) Mice body weight throughout the study protocol. Body weight area under the curve was determined from week 18 to week 24 and compared among the three groups by one-way Anova. (C) Body fat mass at weeks 15,20 and 23 as determined by DEXA analysis. Fat content differences among groups were significant at weeks 20 and 23. (D) Kidney weight at the end of the study. (E) Glycemia and BHB concentration measured in tail blood at week 23. (F) IGTT as performed at week 23 on the three experimental groups. Relevant pairwise statistically significant differences by one-way ANOVA and Tukey's post-hoc test are shown.

Body weight was measured weekly. Dual Energy X-Ray absorptiometry was performed at 15, 20 and 23 weeks of age of the animals using a small-animal densitometer Lunar PIXI (PIXImus™, LUNAR, Madison, WI) to determine the percentage of body fat mass. Blood glucose and BHB levels were measured at week 15 of age (before DMM and change of dietary regimen) and at week 23, at the end of the regimen switch and just before sacrifice after 6 h fasting, using a blood ketone/glucose meter (NovaProTM Glucose/Ketone, Abbot Laboratories, Rungis, France). Intraperitoneal glucose tolerance tests (IGTT) were performed on a separate set of animals submitted to the same protocol, at week 16, prior to dietary switch, and one week before sacrifice. Mice fasted for 6 h (9.00 am–15.00 pm) were injected with a 40% glucose solution in PBS (5 μl/g of body weight) and glycemia was measured at time 0’ (shortly prior to injection) 15′, 30′, 60′, 90′, and 120′ minutes. The study protocol is schematically presented in Figure 1A. The effects of dietary switch on the progression and features of DMM-induced osteoarthritis in the joints will be described elsewhere. After sacrifice, liver and kidney were collected for biochemical analyses.

### 2.2 Lipids extraction and quantification of triglycerides and cholesterol from tissues

Lipids were extracted from 10 mg of liver and kidney tissue previously ground to powder in liquid nitrogen using the Biovision Lipid Extraction Kit (K216-50). The recovered organic phase was evaporated overnight at 37 °C and lipid pellets were brought to a 50 μl volume in suspension buffer, incubated for 20 min at 37 °C in an ultra-sound water bath (Brandson 2510) and stored at −20 °C prior to analysis.

Triglycerides assay was performed on 2 μl of extracted lipids using the Biolabo triglyceride kit (87319) based on colorimetric glycerol-3-phosphate oxidase method that allows linear colorimetric detection of triglycerides at 500 nm at concentrations up to 7.9 mmol/L. Cholesterol was quantified using the Sigma–Aldrich Cholesterol Quantitation kit (MAK043) following the manufacturer's instructions.

### 2.3 Protein extraction, histones acidic extraction and Western blotting

Tissue lysates were prepared by extraction in a lysis buffer (20 mM Tris–HCl, 138 mM NaCl, 2.7 mM KCl, 5% (v/v), glycerol, 1 mM sodium-o-vanadate, 1% (v/v) Nonidet P-40, 5 mM EDTA, 20 mM NaF, 1:1000 proteases inhibitors cocktail (Sigma–Aldrich, P2714) pH 8.0) followed by centrifugation (13,000 g, 15 min, 4 °C) and collection of the supernatant. Residual pellets, containing precipitated chromatin were incubated overnight at 4 °C in 0.2 M HCl to solubilize histones, followed by centrifugation (13,000 g, 15 min, 4 °C). Supernatants were neutralized with 1 M Tris. Protein quantification was performed with the Bradford reagent (BioRad). Tissue lysates were separated on 10% SDS-PAGE and acidic-extracted histones were separated on 15% SDS-PAGE. Standard immunoblotting procedures and ECL detection were employed. Primary antibodies used in this study are listed in Supplementary Table 2.

### 2.4 RNA extraction, reverse transcription and Real-Time quantitative PCR

Total RNA was extracted with the TRI Reagent® (Sigma) according to the manufacturer's instructions. RNA concentration and purity was verified by optical density measurement on a Nanodrop 2000 (Thermo Fisher Scientific).

Reverse transcription was performed using the PrimescriptTM RT reverse transcription kit (Takara) using 1 μg of total RNA in a 20 μl reaction volume. Synthesized cDNA was then diluted to a 1.2 ml final volume with water. qPCR amplification was performed on a Rotor-Gene Real-Time PCR System (Quiagen). Amplification reactions contained 5 μl of cDNA, 5 pmoles of forward and reverse primers and 15 μl of ABsoluteTM QPCR SYBR Green Mix (ABgene). Reactions were incubated at 95 °C for 10 min, followed by 40 cycles of denaturation (95 °C, 10 s), annealing (at gene-specific temperatures for 30 s, see Supplementary Table 3 for primer sequences and annealing temperatures) and elongation (72 °C, 30 s). To verify the amplicon size, qPCR reactions were analyzed on 3% agarose gel. β-actin was used as housekeeping gene for gene expression normalization.

### 2.5 Statistical analysis

Statistical analysis was performed using GraphPad Prism9 (GraphPad Software, USA, www.graphpad.com). Unless stated otherwise in the figure legend, one-way ANOVA, followed by the Tukey's post-hoc multiple comparison test was used to compare the three experimental groups. Statistical significance was set at p < 0.05. Data are presented as mean ± SD. Statistical significances are reported as follows: ∗*p* < 0.05, ∗∗*p* < 0.01, ∗∗∗*p* < 0.001 and ∗∗∗∗*p* < 0.0001.

## 3 Results

### 3.1 After a HFD regimen, chow and ketogenic diets induce weight loss and decrease fat mass, with ketogenic diet inducing a better glycemic normalization

To evaluate the effects of a ketogenic diet on weight loss, 16-weeks old C57Bl6/J mice previously rendered obese by a 10-weeks feeding on a high fat diet were switched to a ketogenic diet (KD group), chow diet (CD group) or kept on a high fat diet (HFD) for 8 weeks (Figure 1A). Following dietary switch, body weight decreased from the first week in both the CD and KD groups as compared to the HFD group, to reach a statistically significant decrease from the second week in the KD group and the third week in the CD group. Weight gain accrued in the HFD group until the end of the protocol and weight loss in the KD and CD groups remained statistically significant until the end of the study (Figure 1B). A significant decrease in fat mass was observed both in the CD and KD group as compared to the HFD group both at 4 and 7 weeks after dietary switch (Figure 1C). Besides the observed loss in fat mass, administration of the KD may also affect internal organs, as the kidney weight was significantly lower in the KD group (Figure 1D). Glycaemia, measured after a 6-hours fasting, was significantly decreased in both CD and KD groups as compared to the HFD group, with stronger glycemic normalization in the KD group showing a return to normoglycaemia (Figure 1E, upper graph). An intraperitoneal glucose tolerance test (IGTT), performed on an independent set of experimental animals, also demonstrated a significantly improved glucose disposal both in the CD and KD group (Figure 1F). As expected, the KD group developed a mild ketosis (Figure 1E, lower graph).

### 3.2 Histone β-hydroxybutyrylation, but not acetylation, is promoted by the ketogenic diet in liver and kidney

**Figure 2:**
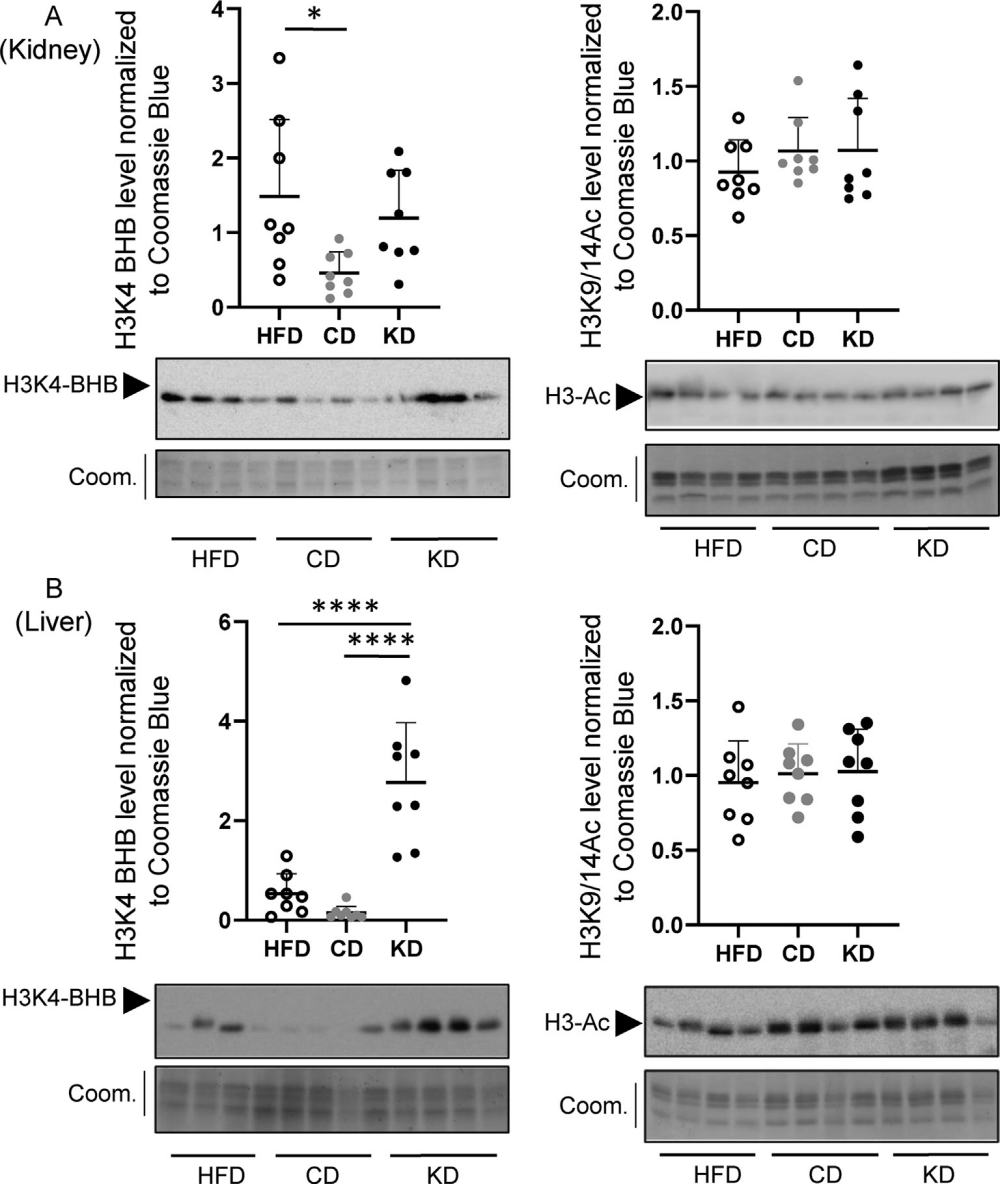
Effects of dietary switch on histone β-hydroxybutyrlation and histone acetylation. Acid-extracted histones from kidney (A) and liver (B) were immunoblotted with antibodies to β-hydroxybutyrylated histone H3 lysine 4 (H3K4–BHB, left graphs) and antibodies to acetylates histone H3 lysines 9/14 (H3-Ac, right graphs). Loading was assessed by coomassie staining. Immunoblotting signal quantification is relative to the coomassie blue signal. Immunoblotting membranes are shown below each graph and in Supplementary Fig. 1 (n = 8 for each experimental condition). All pairwise statistically significant differences by one-way ANOVA and Tukey's post-hoc test are shown.

Histone β-hydroxybutyrylation has been shown to occur in cells treated with BHB [7,8] as well as in the liver of starving mice and mice rendered diabetic by STZ treatment [8]. Here, we evaluated the occurrence of β-hydroxybutyrylation on histone H3 lysine 4 (H3K4–BHB), lysine 9 (H3K9–BHB) and lysine 18 (H3K18–BHB) in the liver and kidney upon administration of a KD. H3K4–BHB and H3K18–BHB were specifically increased in liver (Figure 2 A, B left panels, as well as and Suppl. Figure 1, left panels, and Suppl. Figure 2 A, B right panels), while H3K9–BHB increased in both kidney and liver (Suppl. Figure 2, left panels). When integrating β-hydroxybutyrylation levels on the three lysines by addition of the three independent β-hydroxybutyrylation signals, both organs displayed augmented histone β-hydroxybutyrylation upon dietary switch to the KD as compared to the histone β-hydroxybutyrylation levels in mice on CD or HFD (Supplementary Fig. 1C). Augmented histone β-hydroxybutyrylation in the KD group is a specific chromatin change, as histone acetylation was unaltered in the three dietary conditions (Figure 2A,B right panels and Suppl. Figure 1, right panels).

### 3.3 Strong and specific upregulation of the rate-limiting ketogenic gene *Hmgcs2* in kidney upon ketogenic diet

As β-hydroxybutyrylation, a histone PTM marking gene promoters, is increased in KD-fed mice, we evaluated gene expression levels of ketogenic and ketolytic genes in liver and kidney, including *Hmgcs2* (Hydroxymethylglutaryl-CoA synthase 2), the rate-limiting gene for ketogenesis, and *Oxct1* (3-Oxoacid CoA transferase), the rate-limiting gene for ketolysis.

**Figure 3:**
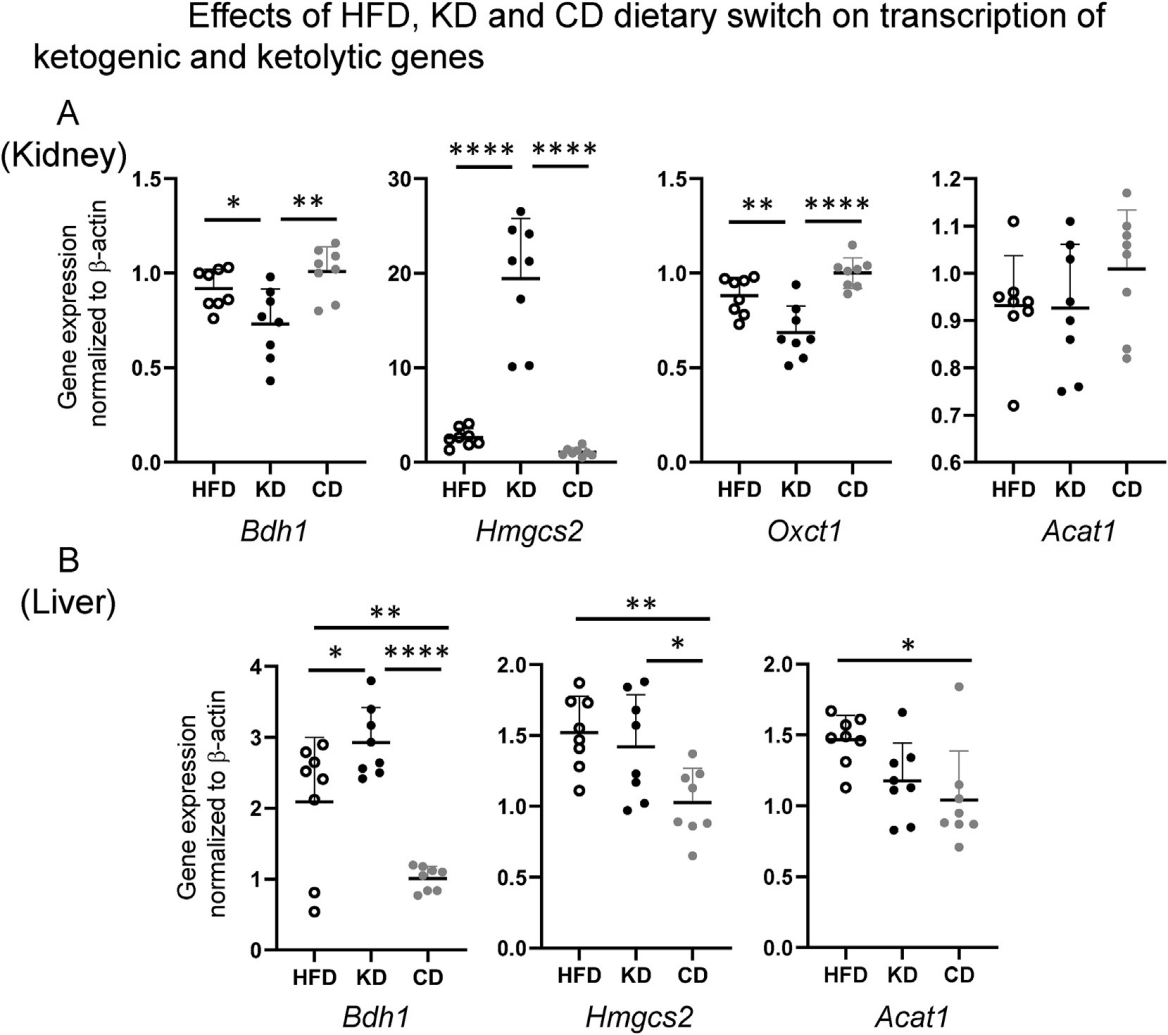
Effects of HFD, KD and CD dietary switch on transcription of ketogenic (*Hmgcs2*) and ketolytic (*Oxct1*) rate limiting genes as well as ketolytic/ketogenic shared genes *Bdh1* and *Acat1* in kidney (A) and liver (B). Detection of cDNAs was performed by RT qPCR and gene expression was quantified by the ΔΔCt method using β-actin as housekeeping gene. All pairwise statistically significant differences by one-way ANOVA and Tukey's post-hoc test are shown.

In kidney, the switch to a KD exerted opposite effects on the expression of ketogenic and ketolytic rate limiting genes *Hmgcs2* and *Oxct1*, with downregulation of ketolytic *Oxct1* and a strong upregulation of the rate-limiting ketogenic *Hmgcs2*, indicating that the kidney is a potentially ketogenic organ under a condition of ketogenic diet feeding (Figure 3A).

In the liver, a switch to a CD led to significant downregulation of ketogenic *Hmgcs2*, compared to both the HFD and KD groups, which was accompanied by a parallel downregulation of *Bhd1* (3-Hydroxybutyrate Dehydrogenase 1) and *Acat1* (Acetyl-CoA acetyltransferase 1) (Figure 3B). In the KD group, neither *Hmgcs2* nor *Acat1* were modulated as compared to the HFD group, while a significant upregulation of *Bdh1* was observed.

### 3.4 Ketogenic diet–induced HMGCS2 protein expression in liver and kidney

**Figure 4:**
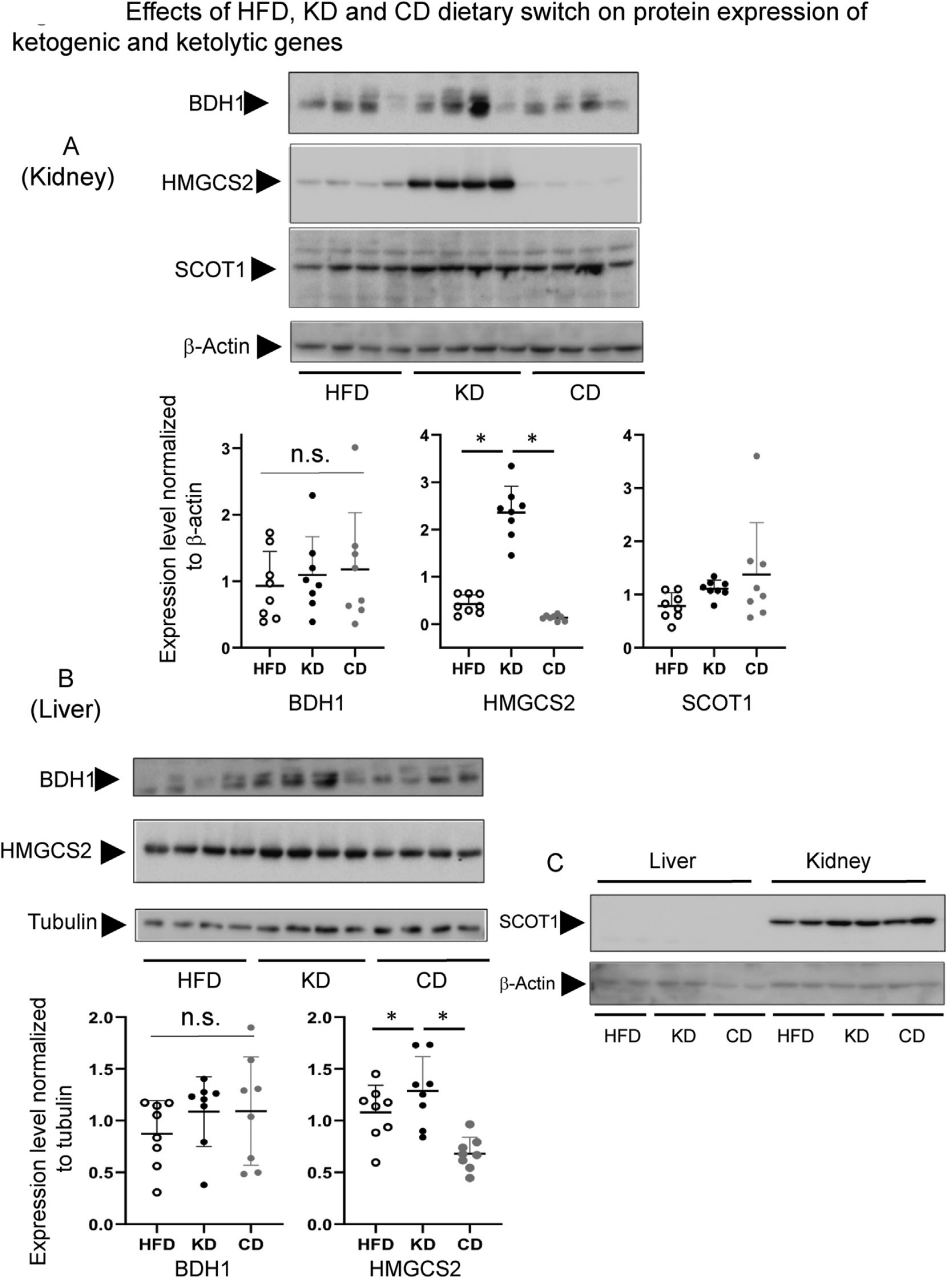
Effects of HFD, KD and CD dietary switch on protein expression of ketogenic HMGCS2, ketolytic SCOT1 (coded by *Oxct1*) and ketolytic/ketogenic BDH1 enzymes in kidney (A) and liver (B). Immunoblotting membranes for (A) and (B) panels are shown in the figure and in Supplementary Fig. 2 (n = 8 for each experimental condition). (C) Direct comparison of SCOT1 expression between liver and kidney. Protein expression was normalized to β-actin or tubulin expression as indicated. In quantification graphs of panel (A) and (B) all pairwise statistically significant differences by one-way ANOVA and Tukey's post-hoc test are shown.

We next determined whether the gene expression alterations observed after the dietary switch are paralleled by protein expression changes. Both in kidney (Figure 4A) and liver (Figure 4B), protein expression of BDH1 displayed a strong individual variability and no differences among dietary conditions. HMGCS2 protein expression levels paralleled gene expression changes in kidney and, to a lesser extent in liver (Figure 4A,B), with the quantitatively stronger upregulation in the kidney of KD animals and a significant increase also in liver. In kidney, protein expression upregulation of the ketolytic rate-limiting enzyme SCOT1 (Succinyl-CoA-Oxoacid Transferase, the protein product of *Oxct1*) was observed in animals switched to a chow diet, in accordance with the upregulation of the corresponding gene *Oxct1* (Figure 4A) while in liver, a purely ketogenic organ, SCOT1 protein expression was undetectable (Figure 4C and Suppl. Fig. 3).

### 3.5 Ketogenic diet induces gene expression adaptations in genes controlling ketogenesis and ketolysis, amelioration of liver lipid load and repression of lipogenic enzymes

**Figure 5:**
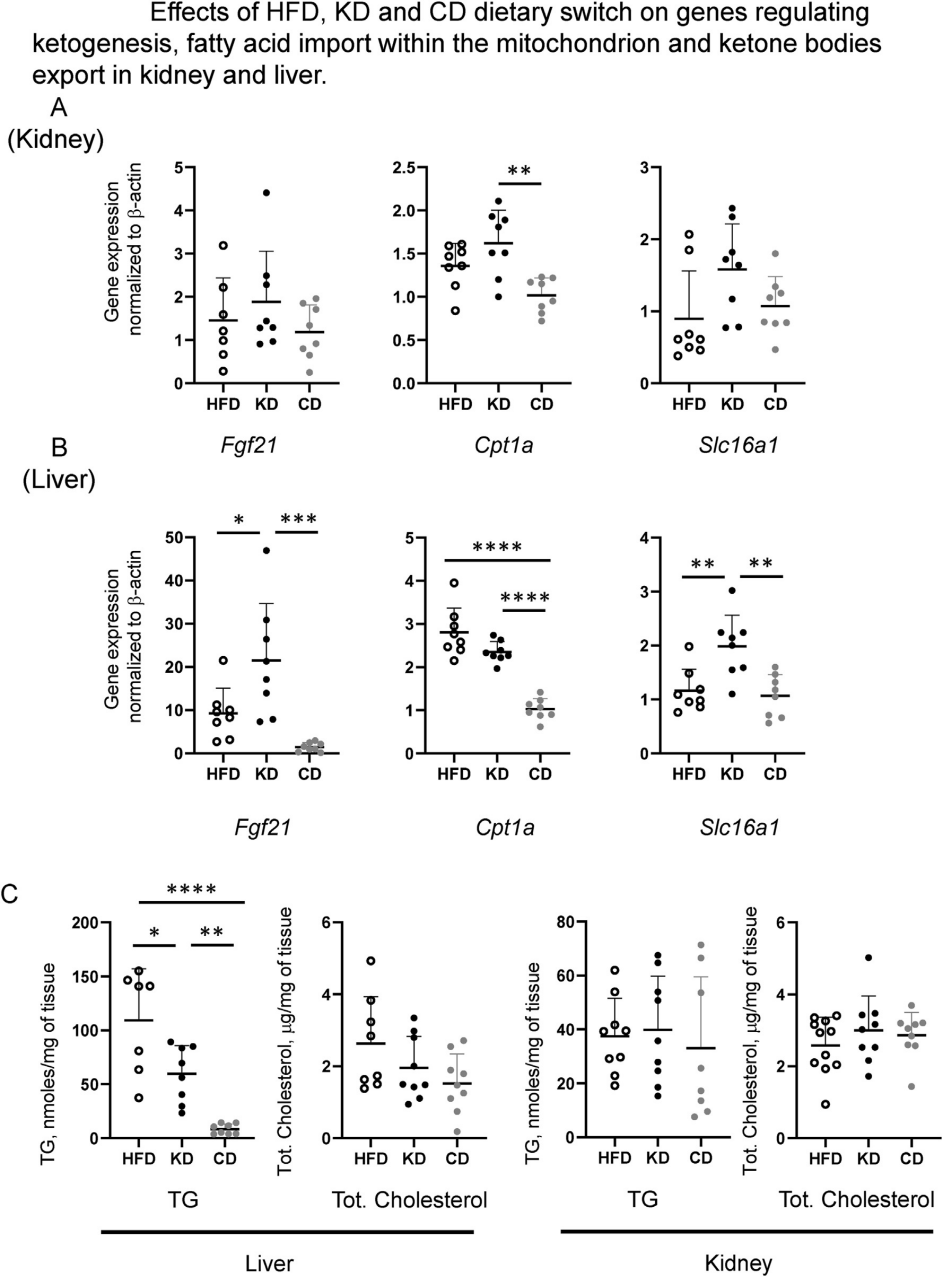
Effects of HFD, KD and CD dietary switch on genes regulating ketogenesis (*Fgf21*), fatty acid import within the mitochondrion (*Cpt1a*) and ketone bodies export (*Slc16a1*) in kidney (A) and liver (B). (C) Quantification of triglycerides and total cholesterol in liver and kidney. All pairwise statistically significant differences by one-way ANOVA and Tukey's post-hoc test are shown.

**Figure 6:**
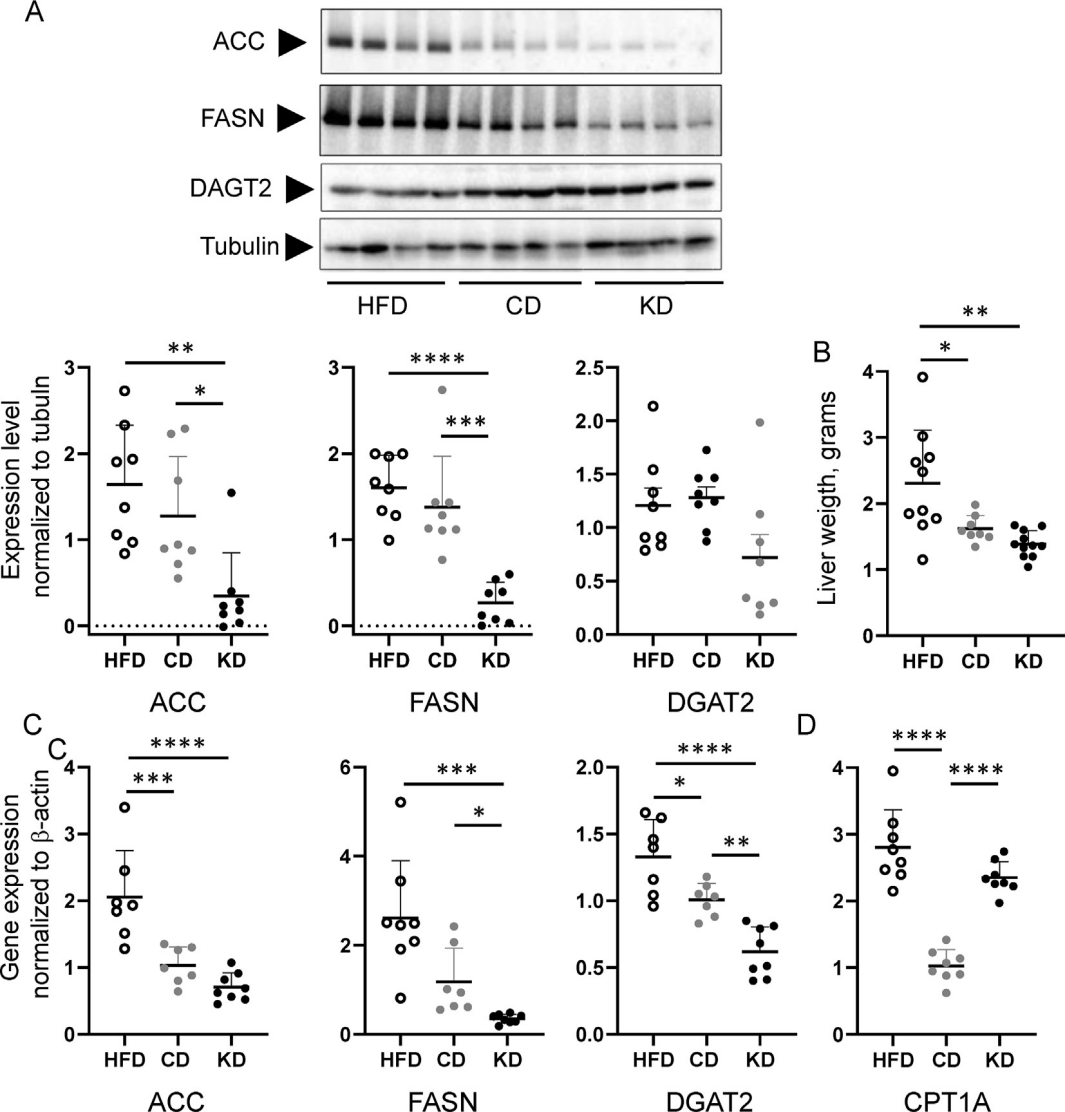
Effects of HFD, KD and CD dietary switch on the expression of lipogenic proteins and genes in the liver. (A) Immunoblotting membranes stained with antibodies directed to ACC, FASN, DGAT2 and tubulin and relative quantifications (normalized to tubulin). Immunoblotting membranes are shown in the figure and in Supplementary Fig. 4 (n = 8 for each experimental condition). (B) Liver weight at the end of the study. (C, D) Gene expression levels of lipogenic (C) and lipolytic (D) genes as performed by RT qPCR. All pairwise statistically significant differences by one-way ANOVA and Tukey's post-hoc test are shown.

Fibroblast growth factor 21 (FGF21) is a metabolic hormone mainly produced by the liver in response to various nutritional conditions that promotes hepatic fatty acid oxidation and ketogenesis [[23], [24], [25]]. In the liver of mice from the KD group, *Fgf21* gene expression was significantly upregulated compared to the HFD and CD groups (Figure 5B). Gene expression of the monocarboxylate transporter *Slc16a1*, allowing the export of ketone bodies, was also increased in the KD group while *Cpt1a* (carnitine palmitoyltransferase 1a), responsible for the transport of fatty acids inside the mitochondrion, while strongly downregulated in the CD group was not altered upon the switch to a KD. In kidney, modifications of genes implicated in the ketogenesis were more unremarkable, with only an upregulation of *Cpt1a* in the KD group as compared to the CD group (Figure 5A). Both dietary switches to the KD and CD led to a significant decrease in hepatic trygliceride content, with a stronger normalization in the CD group (Figure 5C, left graph), while total cholesterol content was not affected in either liver or kidney (Figure 5C, right graphs). Notably, the dietary switch to KD induced, in the liver, a strong repression of the key lipogenic enzymes Fatty Acid Synthase (FASN) and AcCoA carboxylase (ACC) both at the gene and protein level (Figure 6C, A, respectively). Expression of the lipid oxidation gene *Cpt1a* in the KD group remained at levels similar to those of the HFD group, while it dropped for the CD group, likely as a response to the increase in dietary carbohydrates only observed in the CD (Figure 6C). Liver weights were decreased in both CD and KD group as compared to HFD group (Figure 6B).

### 3.6 The Ketogenic diet modulates inflammation markers and insulin signalling in liver and kidney

**Figure 7:**
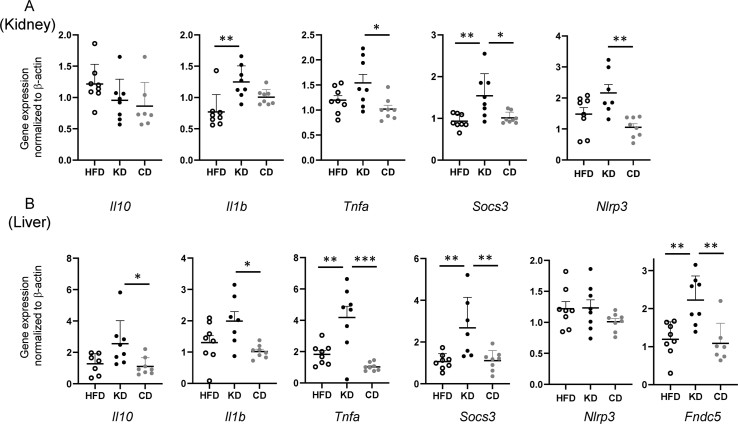
Effects of HFD, KD and CD dietary switch on expression of inflammatory genes. Anti-inflammatory *Il10* and suppressor of cytokine signaling 3 (*Socs3*) and pro-inflammatory *Il1b* and *Tnfa* genes were quantified in kidney (A) and liver (B). Anti-fibrotic gene *Fndc5* was quantified in the liver (B). All pairwise statistically significant differences by one-way ANOVA and Tukey's post-hoc test are shown.

As β-hydroxybutyrate has been reported to be anti-inflammatory, we evaluated the gene expression level of a panel of pro- and anti-inflammatory genes in liver and kidney. After KD dietary switch, increased gene expression of anti-inflammatory cytokine *Il10* was observed in the kidney; in addition, the intracellular suppressor of cytokine signalling 3 (*Socs3*) increased in both liver and kidney (Figure 7). SOCS3, by virtue of its function of STAT3 signalling suppressor may also provide an anti-fibrotic contribution, as STAT3 promotes liver fibrosis [26], and increased gene expression of the anti-fibrotic fibronectin type III domain containing 5 (*Fndc5*, also known as irisin) was observed in the KD group [27] (Figure 7). However, at the same time, the genes for several pro-inflammatory cytokines (*IL1b*, coding for IL-1β; and *Tnfa,* coding for TNFα) and the inflammasome component *Nlrp3* were likewise increased in the KD condition in either or both organs (Figure 7), arguing for an overactivation of part of the inflammatory machinery under a KD.

**Figure 8:**
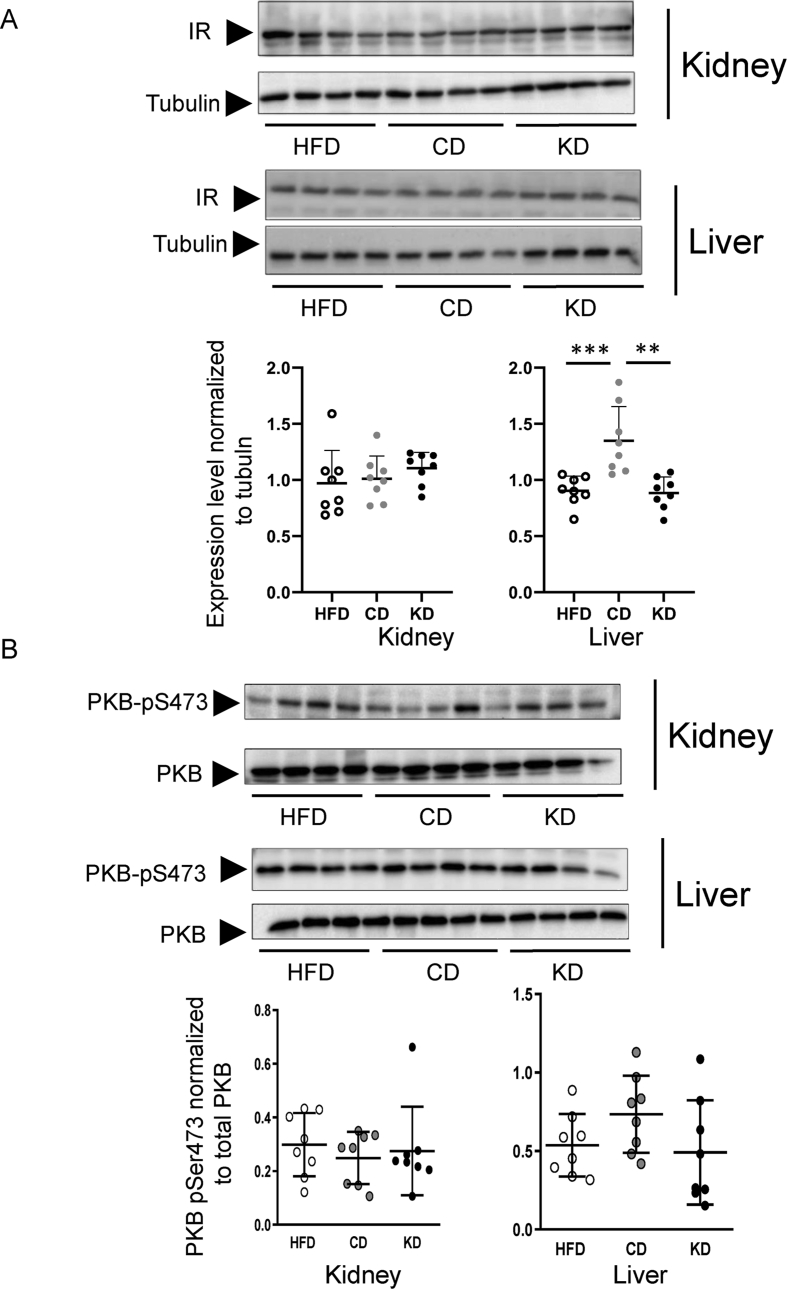
Mild modulation of insulin signalling in the dietary switch to control but not ketogenic diet. The effects of HFD, KD and CD dietary switch on the protein expression of the insulin receptor (IR, panel A); and phosphorylated PKB (on Serine 473, PKB-pSer473, panel B) were evaluated in kidney and liver. Immunoblotting membranes for IR, tubulin, PKB-pSer473 and total PKB are shown in the figure and in Supplementary Fig. 3 (n = 8 for each experimental condition). All pairwise statistically significant differences, determined by one-way ANOVA and Tukey's post-hoc test, are shown.

To further investigate the possible metabolic effects of the KD, we evaluated the protein expression level of the insulin receptor (IR) and the activation status of the key insulin signalling molecule PKB by evaluating its phosphorylation level on Serine 473. A dietary switch to CD, but not to KD, promoted IR overexpression specifically in the liver (Figure 8A). Still, no improvement of PKB activatory phosphorylation was observed (Figure 8B). This may suggest that, at least in the fed state, CD has a minor impact on insulin signalling, while KD has none.

## 4 Discussion

While obesity should not be viewed as a disease *per se*, it is associated to most of the chronic diseases that are major contributors to morbidity and mortality worldwide, such as type 2 diabetes, cardiovascular diseases, hypertension and osteoarthritis [28]. Notwithstanding that the development of obesity carries a non-negligible contribution from non-modifiable hereditary causes [29], numerous studies addressing dietary, pharmacological and lifestyle treatments, alone or in associations, have attempted to solve the simple and urgent problem of inducing weight loss, with short term success [28] often followed by body weight catch up [30]. In recent years, the ketogenic diet has been proposed as a possible nutritional paradigm to reverse obesity. The ketogenic diet, in which calorie intake from dietary carbohydrates is expected to account for <5% of the total calories ingested [31] forces the body to switch to fatty acid metabolism and production of ketone bodies as primary energy source, recapitulating the metabolic features of caloric restriction or fasting. Studies in mouse models have shown that administration of a ketogenic diet reduces mortality and improves cognitive functions [15]; an extension of longevity (akin to caloric restriction) and “health span” was also observed [32].

Here, we attempted to move forward the study of the benefits of a ketogenic diet on weight loss by establishing a protocol in which mice were initially rendered obese by the administration of a high fat diet for 10 weeks and then switched to either a chow diet (CD) or a ketogenic diet (KD) for 8 weeks. An experimental control group submitted to high fat diet for 18 weeks (i.e. without a dietary switch) was also investigated (HFD, Figure 1). All animals, before the dietary switch, were submitted to a DMM surgery to induce osteoarthritis. Osteoarthritis is a common chronic articular disease, particularly prevalent and severe in the obese population. Findings about the effects of the dietary switch on disease progression in this experimental model of obesity-compounded osteoarthritis will be published elsewhere.

The KD and CD were equally effective in reducing body weight and incidence of fat mass. As expected, mice submitted to a KD developed a mild ketosis, with a median concentration of circulating BHB of ≈2 mmol/l. A decreased glycaemia was observed in both the CD and KD groups, with the KD group reverting to an almost normoglycaemic condition. Our study protocol was inspired by a previous study in which a HFD was administered for 12 weeks prior to a switch to a KD or maintenance of a HFD as control [33]. In keep with our data, this study showed that a KD reversed body weight increase in mice previously fed a HFD and [33]. In addition, we showed here that a switch to a KD is metabolically superior because of improved glycaemia, at least during an 8-weeks regimen, to a switch to a chow diet. These results indicate that a KD may be a useful transitory nutritional approach to reverse previously established metabolic disturbances. As a promising clinical translation of our observations, a recent study on obese volunteers with type 2 diabetes demonstrated that a very-low-calorie ketogenic diet was superior to a standard low-calorie diet in inducing glycemic normalization at 3 and 12 month after the start of the dietary treatment [34].

The numerous beneficial effects on inflammation [4,5], metabolism [9], and hypertension [35] of the ketogenic diet may be linked to modulation of gene transcription following histone β-hydroxybutyrylation, an histone PTM promoted by BHB [8]. While global protein β-hydroxybutyrylation has been shown to occur on various organs in mice fed a KD for 4 weeks [9], we provide here, to our knowledge, the first identification of the specific increase of histone β-hydroxybutyrylation in liver and kidney. β-hydroxybutyrylation in skeletal muscle was absent both in CD/HFD groups and in the KD (S. Nasser, unpublished data), suggesting the existence of tissue specificity for this histone PTM. In accordance with this observation, only a small number of genes, mainly belonging to the tricarboxylic acids cycle and cytokine/chemokine signaling were regulated in primary muscle cell cultures exposed to BHB [36]. The ketone body BHB is a precursor for histone β-hydroxybutyrylation [8] and was proposed to be a histone deacetylase inhibitor in the millimolar range when applied to cells in culture [6], a concentration that was reached in the KD group. While increased β-hydroxybutyrylation could be detected in the KD group both in liver and kidney, we could not identify any difference in histone acetylation, suggesting that, *in vivo*, at the low millimolar concentrations characteristic of sustained KD induced ketonemia (∼2 mmol/l), BHB may not be an HDAC inhibitor. Nonetheless, chromatin immunoprecipitation of acetylated lysine on histone H3 from liver and intestine tissues from mice fed a ketogenic diet revealed circadian oscillatory changes of histone acetylation that were higher in amplitude as compared to tissues from mice fed a control diet [37]. Some of the genes that we found to be overexpressed upon the switch to a KD, namely *Socs3* and *Cpt1a*, were similarly upregulated in the liver of mice subjected to a 48 h fasting [8]. Interestingly, these fasted mice displayed an approximately 2-fold increase in histone β-hydroxybutyrylation, but not acetylation, in the vicinity of the TSS of *Socs3* and *Cpt1a* [8]. Other genes, including *Hnf4a*, *Per1* and *Ppargc1b*, however, did not display this correlation (Supplementary Fig. 6). This may suggest that β-hydroxybutyrylation acts as a partly shared transcriptionally-promoting histone PTM between the experimental situation of fasting-induced ketosis [8] and in the KD administered in our study.

Independently from the relative contribution of histone acetylation and histone β-hydroxybutyrylation to the modulation of gene expression, several studies indicate that the ketogenic diet induces extensive transcriptional and proteomic responses that repurpose the body metabolism to cope with the almost total absence of ingested carbohydrates [9,33]. As histone β-hydroxybutyrylation, that we observed upon switch to a KD is an epigenetic regulatory mark linking metabolic adaptations to gene expression, we evaluated the effects of the KD switch on the expression of ketogenic and ketolytic genes in the liver and kidney, representing the quantitatively major ketogenic organ and ketone bodies consuming organ respectively. Higher expression of ketogenic genes both in the HFD and KD condition indicate that the liver contribute to ketogenesis already in the continued HFD feeding condition, but without detectable mild ketosis, irrespective of the presence of dietary carbohydrates. The switch to a KD was, however, affecting the kidney to a greater extent, as we observed an important upregulation of Hydroxymethylglutaryl-CoA synthase 2 (HMGCS2) both at the gene and protein expression level. Gene expression modulation in the liver of *Fgf21* serving as a hormone promoting ketogenesis, *Cpt1a*, allowing fatty acids import in the mitochondria where β-oxidation and ketogenesis take place and the monocarboxylate transporter *Slc16a1*, allowing ketone bodies exit from the cell, all converge to support the notion that transcriptional changes occur in the liver and kidney to adapt the organism to the switch from a HFD to a KD. A recent report using liver- and kidney-specific gene knockout of *Hmgcs2* indicates that renal HMGCS2 overexpression observed in kidney upon fasting does not contribute to whole body ketogenesis [38], while the contribution of hepatic HMGCS2 is essential to avoid the development of non-alcoholic fatty liver disease [39], as HMGCS2 would diminish the lipid load in the liver by redirecting lipids into ketone bodies synthesis. In the switch to a KD diet, we also observed a prominent downregulation of the lipogenic machinery, with substantially decreased gene and protein expression of the rate-limiting lipogenic ACC and FASN which can be explained on the basis that the KD fails to provide lipogenic substrates – i.e. carbohydrates.

Our results support the possibility that nutrition based on a ketogenic diet may, at least in the short term, lead to successful weight loss with improvement of metabolic parameters in obese individuals. Recently, a small scale randomized controlled trial evaluating the effects of a low-carbohydrate (<50 g/day) and energy-restricted diet in mildly obese diabetic patients for 12 weeks demonstrated improvement of multiple anthropometric and clinical parameters, including decreased body weight, BMI and fat mass, glycaemic normalization, and decreased systolic and diastolic blood pressure, accompanied with a reduction – to full discontinuation in some participants – of antidiabetic medications [40]. Similarly, a less stringent approach with a dietary reduction of caloric carbohydrate intake from 50% to 30% for 6 weeks showed a mild improvement of glycemic control and decreased circulating and hepatic triglycerides in mildly obese type 2 diabetic patients, although ketosis was unlikely to develop [41].

Administration of a ketogenic diet has shown beneficial effects, at least in the short time course of the dietary intervention in women suffering of polycystic ovary syndrome (PCOS), the most common endocrine disorder of women at reproductive age, associated with obesity, hyperinsulinemia, and insulin resistance [42]. In a pilot study on 11 women, administration of a very low calories ketogenic diet for 24 weeks led to significant body weight decrease and improvements of fasting insulin [43]. Similarly, Cincione et al., administered a ketogenic diet to 17 obese women with PCOS, observing the induction of ketosis and improvement of anthropometric and biochemical parameters including an improvement of the LH/FSH ratio, insulin sensitivity and HOMA index [44]. Whether modulation of inflammatory responses is critically involved in the effects of KD is still a matter of debate. While earlier studies in mice models supported the establishment of an anti-inflammatory state [4,5], we rather observed a general immune activation, with KD-inducing an increase of both pro- and anti-inflammatory factors. Our observation is in line with a recently published study on a small cohort of obese patients receiving a very-low calories ketogenic diet in which an immunomodulatory effect with increases in both pro- and anti-inflammatory factors was observed [45].

Despite its novelty in assessing the effects of a KD after induction of obesity by prior administration of a HFD, our study presents some limitations. Firstly, prior to the dietary switch from HFD to a KD or CD, all experimental mice have been subjected to a destabilization of the medial meniscus (DMM), a surgical model to induce osteoarthritis that, although applied to all animals, may have had confounding effects on metabolism. Secondly, our study protocol permitted *ad libitum* feeding, preventing us to define the relative contribution of food intake versus metabolic adaptations in the normalization of body weight, fat mass and triglycerides content normalization observed in the KD and CD groups. Finally, mice receiving the ketogenic diet, which contains 6% of available energy from proteins as compared to 20% in the HFD and CD, simultaneously experience dietary protein restriction which, together with the carbohydrate virtual absence and fat overrepresentation, could contribute to the observed results.

In conclusion, our findings indicate that in a model of body weight loss the ketogenic diet can be, in the short term, superior to a switch to a normal diet (chow diet in mice). Among the specific adaptations and responses elicited by the KD we observed an improved glycemia, lowered mass of kidney weight and occurrence of specific histone β-hydroxybutyrylation as a histone PTM potentially governing KD-induced gene and protein expression modulation.

## Author contributions

LP and MS conceived the work, performed experiments, wrote manuscript. SN, TS and NV performed experiments. LP, MS, TT, AB secured funding. All authors analyzed data, discussed research strategies and commented on the developing manuscript versions.

## Data Availability

Data will be made available on request.
