# Supplementary material for: Ketogenic diet administration to mice after a high-fat-diet regimen promotes weight loss, glycemic normalization and induces adaptations of ketogenic pathways in liver and kidney
Source: Mol Metab. 2022 Aug 20;65:101578. doi: 10.1016/j.molmet.2022.101578 (PMC9460189; doi:10.1016/j.molmet.2022.101578)
Supplement: Multimedia component 1 [file mmc1.pptx]

## Slide 1
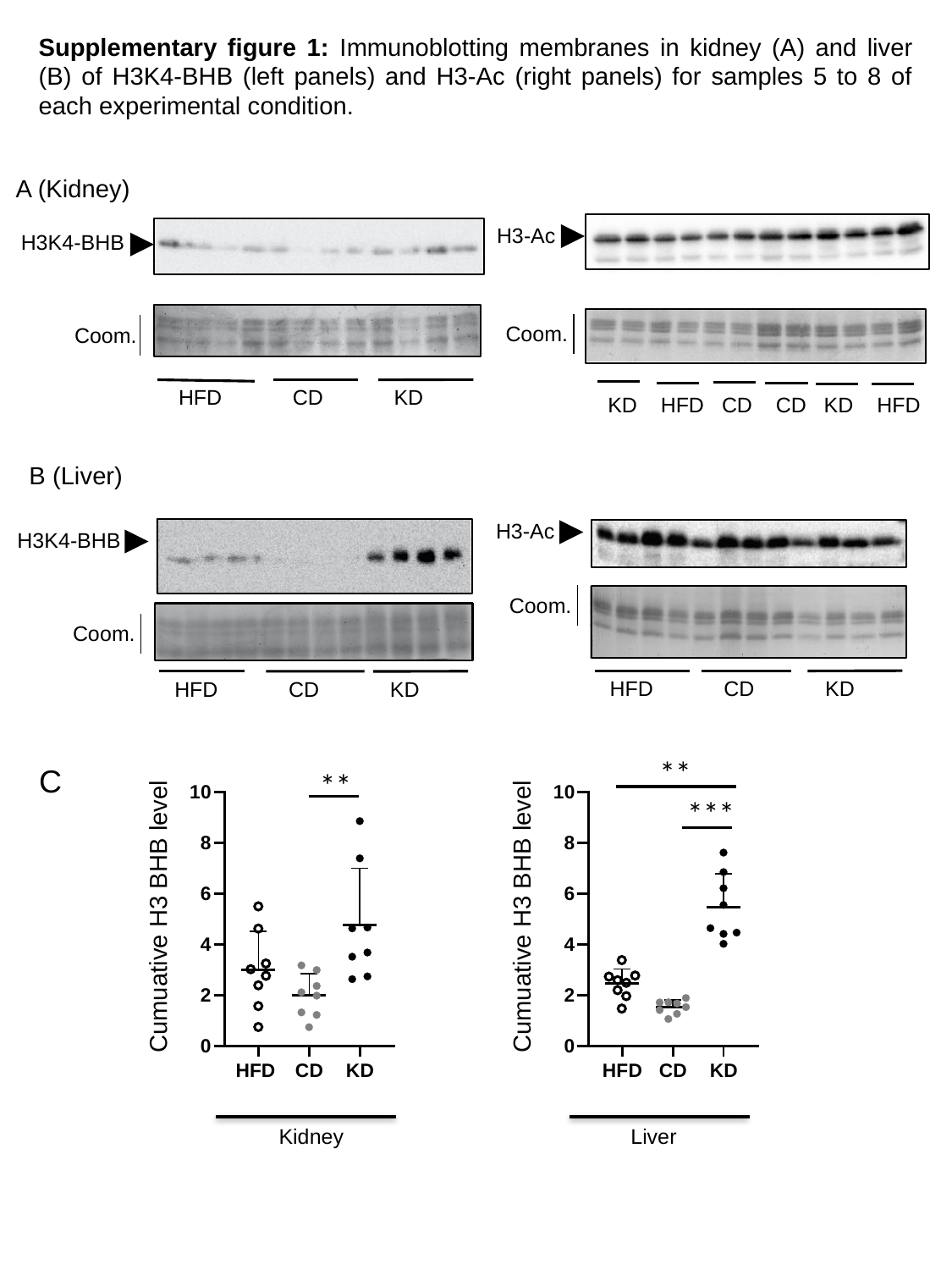

Supplementary figure 1: Immunoblotting membranes in kidney (A) and liver (B) of H3K4-BHB (left panels) and H3-Ac (right panels) for samples 5 to 8 of each experimental condition.
A (Kidney)
H3-Ac
H3K4-BHB
Coom.
Coom.
 HFD CD KD
 KD HFD CD CD KD HFD
B (Liver)
H3-Ac
H3K4-BHB
Coom.
Coom.
 HFD CD KD
 HFD CD KD
**
C
Cumuative H3 BHB level
Kidney
Cumuative H3 BHB level
Liver
**
***

## Slide 2
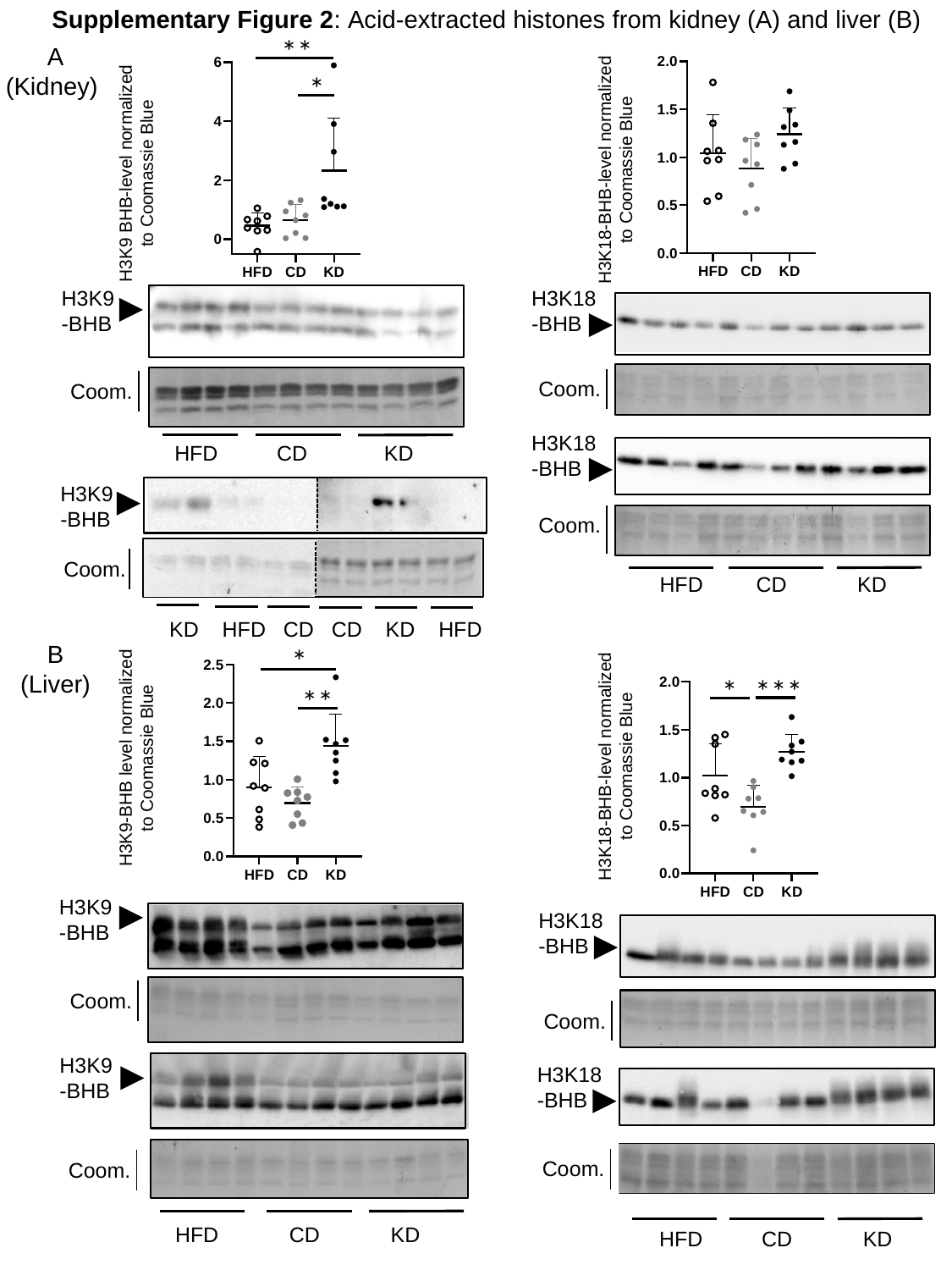

Supplementary Figure 2: Acid-extracted histones from kidney (A) and liver (B)
**
A
(Kidney)
*
H3K18-BHB-level normalized
 to Coomassie Blue
H3K9 BHB-level normalized
 to Coomassie Blue
H3K9
-BHB
H3K18
-BHB
Coom.
Coom.
H3K18
-BHB
HFD CD KD
H3K9
-BHB
Coom.
 KD HFD CD CD KD HFD
Coom.
 HFD CD KD
B
(Liver)
*
***
*
**
H3K9-BHB level normalized
 to Coomassie Blue
H3K18-BHB-level normalized
 to Coomassie Blue
H3K9
-BHB
H3K18
-BHB
Coom.
Coom.
H3K9
-BHB
H3K18
-BHB
Coom.
Coom.
 HFD CD KD
 HFD CD KD

## Slide 3
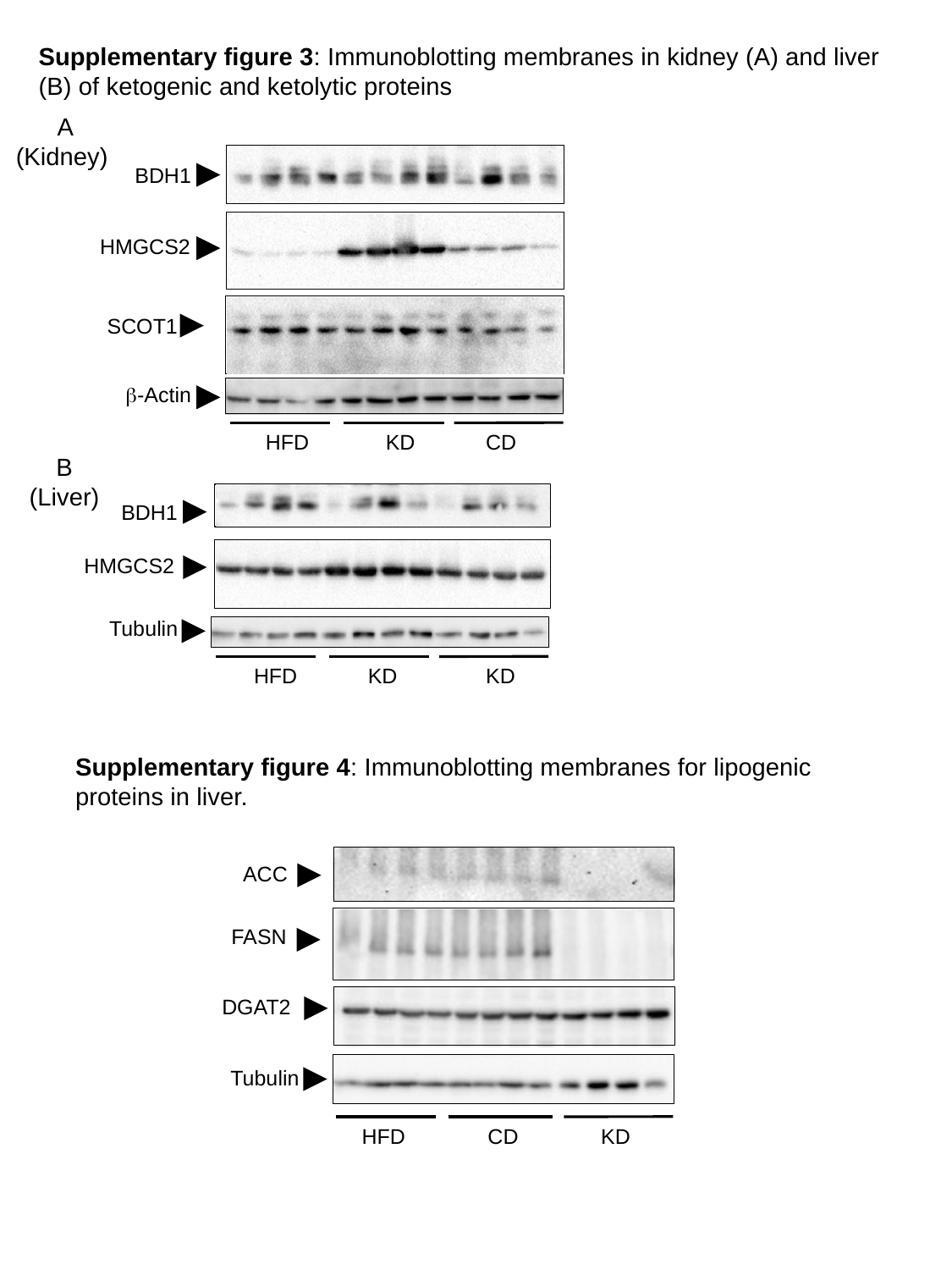

Supplementary figure 3: Immunoblotting membranes in kidney (A) and liver (B) of ketogenic and ketolytic proteins
A
(Kidney)
BDH1
HMGCS2
SCOT1
b-Actin
 HFD KD CD
B
(Liver)
BDH1
HMGCS2
Tubulin
 HFD KD KD
Supplementary figure 4: Immunoblotting membranes for lipogenic proteins in liver.
ACC
FASN
DGAT2
Tubulin
 HFD CD KD

## Slide 4
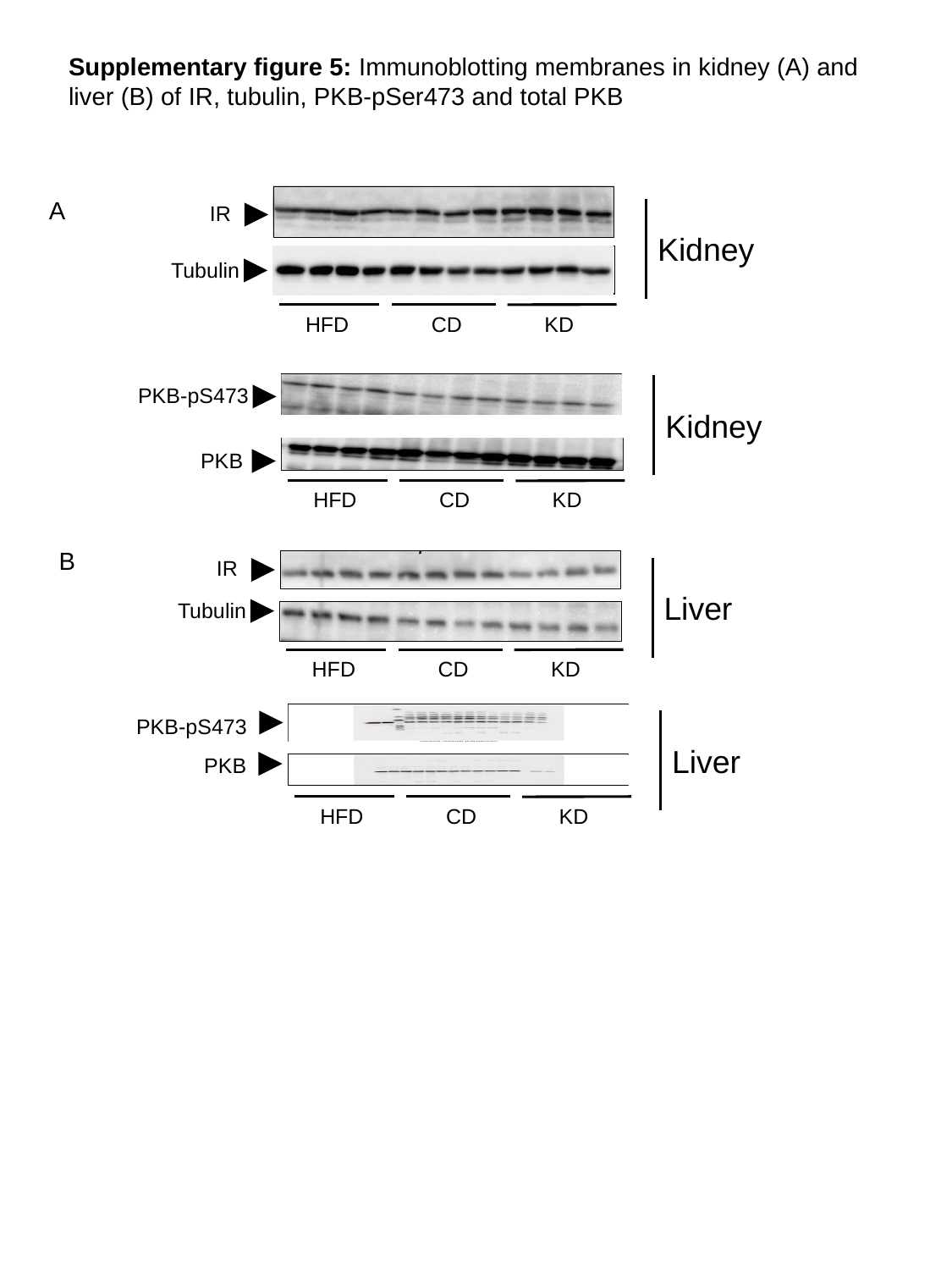

Supplementary figure 5: Immunoblotting membranes in kidney (A) and liver (B) of IR, tubulin, PKB-pSer473 and total PKB
A
IR
Kidney
Tubulin
 HFD CD KD
PKB-pS473
Kidney
PKB
 HFD CD KD
B
IR
Liver
Tubulin
 HFD CD KD
PKB-pS473
Liver
PKB
 HFD CD KD

## Slide 5
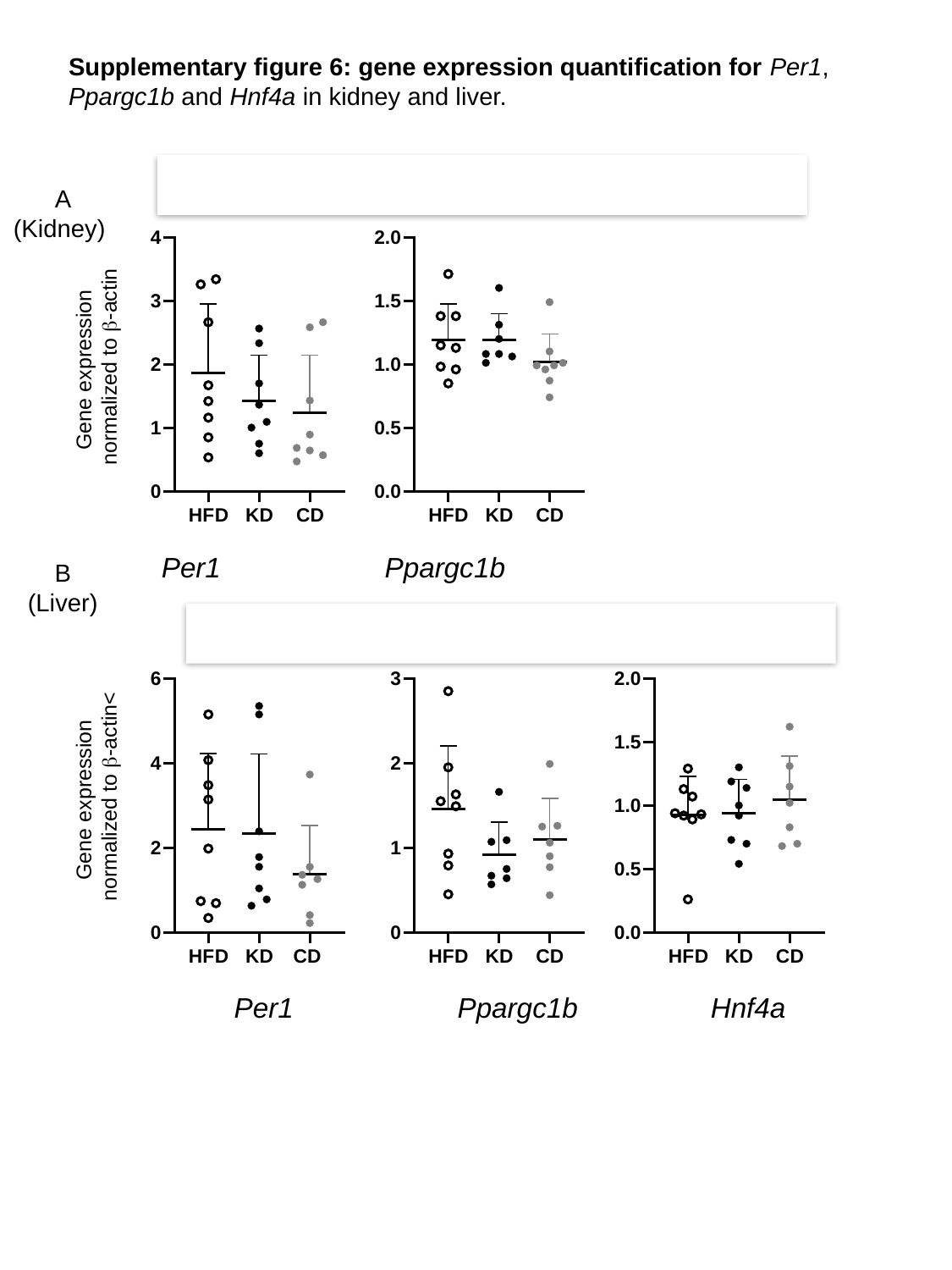

Supplementary figure 6: gene expression quantification for Per1, Ppargc1b and Hnf4a in kidney and liver.
A
(Kidney)
Gene expression
 normalized to b-actin
Per1 Ppargc1b
B
(Liver)
Gene expression
 normalized to b-actin<
Per1 Ppargc1b Hnf4a
