# Supplementary material for: Ketogenic diet administration to mice after a high-fat-diet regimen promotes weight loss, glycemic normalization and induces adaptations of ketogenic pathways in liver and kidney
Source: Mol Metab. 2022 Aug 20;65:101578. doi: 10.1016/j.molmet.2022.101578 (PMC9460189; doi:10.1016/j.molmet.2022.101578)
Supplement: Multimedia component 2 [file mmc2.docx]

1. **Supplementary table 1 (related to materials and Methods):** Macronutrient composition of
2. the diets used in this study 3

| **Diet** | **High Fat Diet, HFD** | **Ketogenic diet, KD** | **Chow diet, CD** |
| --- | --- | --- | --- |
| **Provided by (Company Name)** | SSNIFF  Spezialdiäten, Soest, Germany | SSNIFF Spezialdiäten, Soest, Germany | SAFE (Scientific Animal Food & Engineering), Auge, France |
| **Catalog Number** | EF acc. D12492 | EF R/M with 80% Fat | U8200G10R |
| **Gross energy content (Mj/kg)** | 24,4 | 34,9 | 14,21 |
| **% of energy provided by fats** | 60 | 94 | 13,5 |
| **% of energy provided by proteins** | 19 | 6 | 25,2 |
| **% of energy provided by carbohydrates** | 21 | < 1% (Sugar content is 0,7 % w/w in this diet) | 61,3 |

4

5

1 **Supplementary table 2 (related to Materials and Methods):** Antibodies used in this study 2

| **Antibody** | **Catalog Number** | **Provider** | **Raised in** | **Protein size on western**  **blot** | **Dilution** |
| --- | --- | --- | --- | --- | --- |
| **Anti BDH1** | 514413 | Santa Cruz | Mouse | 33 Kda | 1/2000 |
| **Anti HMGCS2** | ab137043 | Abcam | Rabbit | 50 Kda | 1/2000 |
| **Anti SCOT1** | ab241125 | Abcam | Rabbit | 56 Kda | 1/10000 |
| **Anti FASN** | sc20140 | Santa Cruz | Rabbit | >250 Kda | 1/2000 |
| **Anti ACC** | 36625 | Cell Signalling Technologies | Rabbit | >250 Kda | 1 /2000 |
| **Anti DGAT** | 293211 | Santa Cruz | Rabbit | 40 Kda | 1 /2000 |
| **Anti IR β-subunit** | 06-492 | Upstate Millipore | Rabbit | 75 Kda | 1/5000 |
| **Anti Total Histone H3** | ab1971 | Abcam | Rabbit | 17 Kda | 1/2000 |
| **Anti Acetylated H3 K9/14** | sc8655 | Santa Cruz | Rabbit | 17 Kda | 1/1000 |
| **Anti β-hydroxybutyrylated Histone H3 (Lys9)** | PTM1250 | PTM Biolabs | Rabbit | 15 Kda | 1/2000 |
| **Anti β-hydroxybutyrylated Histone H3 (Lys4)** | PTM1258 | PTM Biolabs | Rabbit | 15 Kda | 1/2000 |
| **Anti β-hydroxybutyrylated Histone H3 (Lys18)** | PTM1252 | PTM Biolabs | Rabbit | 15 Kda | 1/2000 |
| **Anti α-Tubulin** | T5168 | Sigma | Mouse | 50 Kda | 1/2000 |
| **Anti β-Actin** | A5060 | Sigma | Rabbit | 42 Kda | 1/2000 |
| **Anti-Mouse IgG (H+L)-HRP Conjugated** | 172-1011 | Biorad | Goat | - | 1/10000 |
| **Anti-Rabbit IgG (H+L)-HRP Conjugated** | 172-1019 | Biorad | Goat | - | 1/5000 |

3

4

5

6

7

8

1 **Supplementary table 3 (related to Materials and Methods):** Primers used in this study 2

| **Gene name** | **Primers (S, sense; AS, antisense)** | **Gene name (protein name if different)** | **Ref seq number** | **Ampli con size, bp** | **Anneali ng temper ature** |
| --- | --- | --- | --- | --- | --- |
| Acetyl-Coenzyme A acetyltransferase 1 | S :AGACATTGCCATGTGGGAAG AS:CAAATACTAGCCAGACCGAAC | *Acat1* | NM_144784.3 | 204pb | 60°C |
| Beta actin | S :AGCCATGTACGTAGCCATCC AS:TCCCTCTCAGCTGTGCTGGTGAA | *Actb* | NM_007393 | 231pb | 60°C |
| 3-hydroxybutyrate dehydrogenase, type 1 | S :GTTAACAACGCAGGCATCTC AS:AACTTGGTGATGCAGTATGG | *Bdh1* | NM_00112268 3.1 | 215pb | 60°C |
| carnitine palmitoyltransferase 1a | S :ACGTATGAGGCTTCCATGAC AS:GGTGAGTCGACTGCCAGATA | *Cpt1a* | NM_013495 | 243pb | 60°C |
| Fibroblast growth factor 21 | S :GGTACCTCTACACAGATGAC AS:AAGTGAGGCGATCCATAGAG | *Fgf21* | NM_020013 | 208pb | 60°C |
| 3-hydroxy-3- methylglutaryl-Coenzyme A synthase 2 | S :GCTGCCAACTGGATGGAG AS:GTCGTACGCGTTCTCCATGT | *Hmgcs2* | NM_008256 | 195pb | 60°C |
| Peroxisome proliferator activated receptor alpha | S :GAGAATCCACGAAGCCTAC AS:GAAGAATCGGACCTCTGCCTC | *Pparα* | NM_011144 | 196pb | 60°C |
| Interleukin 10 | S :CAGGGCCCTTTGCTATGGTG AS:CGGCTGGGGGATGACAGTAG | *Il10* | NM_010548 | 168pb | 58°C |
| 3-oxoacid CoA transferase 1 | S :GTTGACAACTTCGGCCTGG AS:CCAGAGTCCCATACCCTGTG | *Oxct1*  (SCOT1) | NM_024188.6 | 214pb | 55°C |
| Solute carrier family 16  (monocarboxylic acid transporters), member 1 | S :TGCTTGCCCCTTTGTCTAC AS:ACAGGGCAGCATTCCACAA | *Slc16a1*  (MCT1) | NM_009196 | 172pb | 60°C |
| Sirtuin 3 | S :CCGACATTGTGTTCTTTGG AS:TCAAGCTGGCAAAAGGCTC | *Sirt3* | NM_00117780 4 | 126pb | 60°C |
| Interleukin 1β | S :ACTGTTCCTGAACTCAACTG AS:CTTGTTGATGTGCTGCTGCG | *Il1β* | NM_008361 | 168pb | 60°C |
| NLR family, pyrin domain containing 3 | S :ACCTGGGCAACAATGATCTT AS : CACGCCTACCAGGAAATCTC | *Nlrp3* | NM_145827, | 193pb | 60°C |
| Tumor necrosis factor α | S :CCAGACCCTCACACTCAGATC AS:CACTTGGTGGTTTGCTACGAC | *Tnfa* | NM_013693 | 79pb | 58°C |
| Suppressor of cytokine signalling 3 | S :CCACGGAACCCTCGTCCGAAGT AS:GTAGTAAGCTCTCTTGGGGG | *Socs3* | NM_007707 | 80pb | 60°C |
| Fatty acid synthase | S : CTGTGCTTGCAGCTTACTGG AS: ACGGTGTCCTCAGAGTTGTG | *Fasn* | NM_07988 | 151pb | 60°C |
| AcetylCoA carboxylase | S : GAGCAAGGGATAAGTTTGAG AS: AGGTGCATCTTGTGATTAGC | *Acaca* | NM_133360 | 133pb | 58°C |

| Diacylglycerol acetyltransferase | S :TGGGTCCAGAAGAAGTTCCAGAA GTA  AS:ACCTCAGTCTCTGGAAGGCCAAA T | *Dgat2* | NM_007707 | 80pb | 60°C |
| --- | --- | --- | --- | --- | --- |
| Carnitine palmitoyltransferase 1a | S : ACGTATGAGGCTTCCATGAC AS: GGTGAGTCGACTGCCAGATA | *Cpt1a* | NM_013495 | 243pb | 60°C |
| Fibronectin type III domain containing 5 | S : CTCTCAGCAGAAGAAGGATG AS: ACCACAACAATGATCAGCAC | *Fndc5* | NM_027402 | 282pb | 60°C |
| Hepatocyte nuclear factor 4A | S : GTGTGAGTCTATGAAGGAGC  AS: TGTAGTCATTGCCTAGGAGC | *Hnf4a* | NM_008261 | 188pb | 58°C |
| Period 1 | S : TGAGCCAGAGGCCAGATTG  AS: TGTATGGCTGCTCTGACTG | *Per1* | NM_0011065 | 229pb | 60°C |
| Peroxisome proliferator-activated  receptor-gamma coactivator-1 beta | S : GGAAGCGGCGGGAAAAGGCC  AS:GCTGTCAAAATCCATGGCTTC | *Ppargc1b* | NM_133249 | 368pb | 58°C |

1

2

3

4
